# Effect of Psychoeducation Group Training Based on Problem-Solving Skills for Women Experiencing Bipolar Spouse Abuse

**DOI:** 10.3389/fpubh.2021.561369

**Published:** 2021-04-21

**Authors:** Maryam Seyyedi Nasooh Abad, Saeed Vaghee, Seyedeh Zahra Aemmi

**Affiliations:** ^1^Department of Psychiatric Nursing, School of Nursing and Midwifery, Mashhad University of Medical Sciences, Mashhad, Iran; ^2^Nursing and Midwifery Care Research Centre, School of Nursing and Midwifery, Mashhad University of Medical Sciences, Mashhad, Iran; ^3^Department of Nursing, School of Nursing and Midwifery, Mashhad University of Medical Sciences, Mashhad, Iran

**Keywords:** bipolar disorder, Iran, problem-solving, spouse abuse, women

## Abstract

**Aim:** Wives of patients with bipolar disorder as informal caregivers are at high risk for spouse abuse and need to learn coping strategies such as problem-solving skills to manage problematic situations. The purpose of this study was to assess the effectiveness of the psychoeducation group training based on problem-solving skills for women experiencing bipolar spouse abuse.

**Methods:** A randomized clinical trial design was used for this study. In intervention group, women experiencing bipolar spouse abuse participated in four problem-solving skills training sessions and women in two groups (intervention = 30 and control = 30) completed the Index of Spouse Abuse at baseline and after 2 months.

**Results:** The results indicated that changes in abuse scores (physical, non-physical and total of Index of Spouse Abuse) after the intervention were significantly different among the two groups (*p* < 0.0001). Although abuse scores decreased in both the intervention and the control groups, but lower abuse scores in the intervention than control group were statistically significant.

**Conclusion:** Our findings support that the problem-solving skills training intervention can help to decrease the women experience of bipolar spouse abuse.

## Introduction

Based on the results of systematic review in 2020, the prevalence of mental disorders in Iran's general population was reported 25.42−31.03% (1). Bipolar disorder (BD), as a chronic, severe and recurrent mental disorder was considered as the sixth leading cause of disability among the physical and psychiatric disorders (2). Bipolar I disorder (BPD-I) is characterized by episodes of depression and mania/hypomania, the severely debilitating symptoms of which can have profound life-long adverse effects on the patient's mental and physical health, educational and occupational functioning, and interpersonal relationships (3–6).

Although based on epidemiological studies, bipolar I disorder is considered to be approximately equally common in men and women, there are several of the differences associated with gender in bipolar patients. Predomination of depressive features in women and manic features in men, difference in regards to psychiatric and medical comorbidities (more endocrine/metabolic disorders in women and more neurological and cancer disorders in men) are mentioned as some of these differences (7).

Also, these patients have problems and challenges in their family function and relationship and the result of review suggests that patients with BD experience multiple negative marital and sexual consequences in their life (5). Family members of the BD patient relatively experience many difficulties, stress and pressures during the episode of the illness, treatment, rehabilitation, and recovery and there is a fear of new relapses, even when the illness is stabilized (2, 4).

Wives of BD patients have a caregiver role and provide social support for these patients (5). This high caregiver burden is very demanding, frequently distressing, and harmful to health and quality of life that wives may experience spousal abuse and violence during living with these mental disorder patients (2, 8).

Abuse as a social and legal issue and global public health problem, is commonly defined as “a single, or repeated act, or lack of appropriate action, occurring within any relationship where there is an expectation of trust which causes harm or distress to a person” (9) and a pattern of abusive behaviors is intimate partner violence (IPV) (sexual, physical or psychological) (10).

Most of the evidence to date suggests mental disorders are considered as one of the risk factors for IPV perpetration against women and have reported an increased risk of IPV perpetration among individuals with mental disorder) two to eight times compared with the general population) (11). Finding of a systematic review demonstrated the men and women with psychiatric disorder have an increased risk of having violence to partners, but more so for men than for women (12).

The physical and mental health of victims is negatively affected by abuse and affected women may experience the chronic somatic complaints, reproductive problems, injuries (13), depression, post-traumatic stress disorder, anxiety, suicidal behavior, substance use, sleep and eating disorders, feelings of guilt, shame and of being worthless (13–17). The negative effects of spousal abuse are intergenerational in that maternal abuse is associated with risk for poor behavioral functioning in her child (16).

In prior research, the illiteracy, low income, lower age at marriage, shorter duration of marriage, physical disease, mental and psychiatric disorder, and substance use were considered as risk factors of domestic violence in Iranian families (18). In order to protect women in relationships with a mental disorder spouse, they need to be clearly and accurately identified and educated (15, 19).

Mental health nurses have an important role in the assessment, identification, management and safety planning for affected women who may be at risk for violence and abuse by the bipolar spouse (20). The result of a systematic review revealed different psychosocial interventions in which psychoeducation is one of their core and main components demonstrating a promising results in Iranian mental health research (21).

Also, use of some coping strategies such as problem-solving method in psychoeducation, as an essential component of cognitive-behavioral therapy, can enable an individual to manage the problematic situations of everyday life and his/her emotional impact. This method can help the individual to find effective or adaptive solutions for his/her daily life problems (22).

Recent evidence suggests that the use of interventions to address problem-solving skills for abused women, may be beneficial in increasing their abilities to navigate the daily stressors of life following abuse (23). Also, the result of a systematic review demonstrated that interventions focused on some strategies such as problem-solving lead to reductions in victimization and offer promise in facilitating and maintaining positive physical and mental health and quality of life changes for women who experienced violence (24).

Abuse and violence against women, as a public health priority and worldwide concern are common that need to the international and interdisciplinary attention and intervention. Prevalence rates of spouse abuses are significantly higher in mental health disorder patients, but the studies are limited. Previous work has identified problem-solving skills as an essential intervention to counter with abuse and violence that nurses can use this intervention to provide better care for affected individuals. However, current literature does not address the role of nurses in this area.

This study aims to explore at baseline and at 2 months, the effectiveness of problem-solving skills group training on spouse abuse scores in women who experienced bipolar spouse abuse. We hypothesized that psychoeducation group training based on problem-solving skills can help to decrease the women experience of bipolar spouse abuse.

## Materials and Methods

### Subjects and Study Design

The present research was a pilot psychoeducation intervention with a controlled group in which a total of 60 wives of patients with bipolar I disorder hospitalized in Ibn-e-Sina hospital in Mashhad in 2017–2018 (October-May) took part. Ibn-e-Sina hospital is a subspecialty Medical Center Governmental referral and teaching hospital for psychiatric patients in Mashhad, Iran, affiliated to the Mashhad University of Medical Sciences. Women who had a spouse's physical abuse score more than 10, or the non-physical spouse abuse score >25 from the Index of Spouse Abuse (ISA) (25) and met the inclusion criteria were randomly assigned to intervention and control groups.

Confirmed diagnosis of bipolar I disorder for hospitalized spouse, lack of speech and hearing problems, no history of drug abuse, not having a history of participation in the problem-solving skills training workshop, live with her spouse (at the time of the study), at least 1 year passed since her marriage time, be the only legal wife of patient, no history of disabling and major medical and psychological problems were considered as inclusion criteria for women. Women who were absent from training program more than one session, those who did not perform home practice and did not complete the *post-test* and those who were living separately from their spouse during the 2 months after the spouse's discharge until the *post-test* time, were excluded from the study.

The sample size was calculated according to the mean comparison formula in two independent samples in this study. Estimation of standard deviation was obtained from a pilot study with 15 participants (per group). According to the formula, the sample size for each group was 25 samples, which was considered taking into account the probability of loss, 30 samples in each group. Z_1_ = 95% = 1.96; Z_2_ = 80% = 0.84 (test power); s and m represent estimated values of standard deviation and mean of spouse abuse in the groups, respectively. The pilot study samples were not included in the main study.

**Figure 1:**
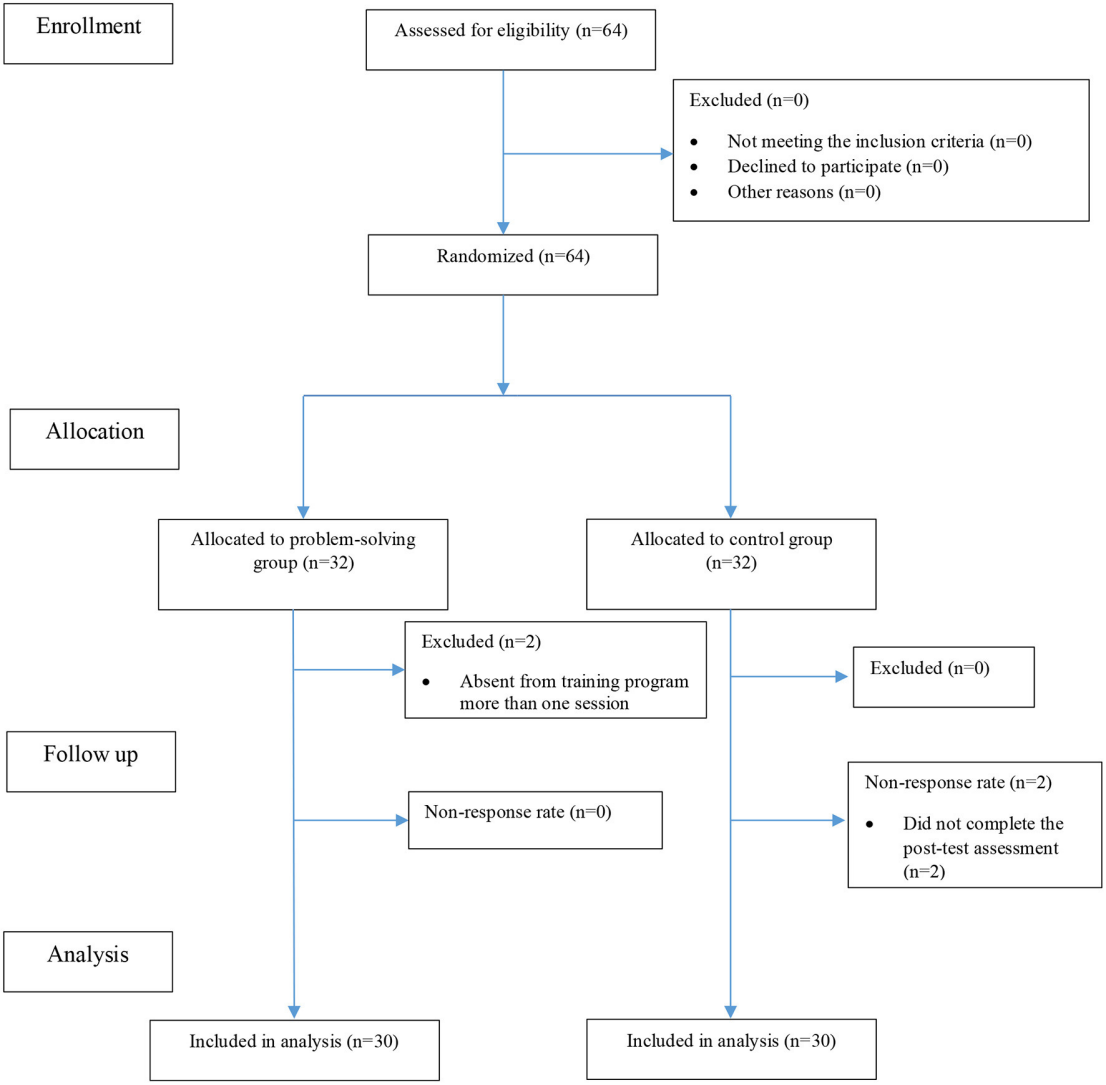
Flow diagram for the sampling procedure.

A total of 60 women were selected and divided with simple random allocation method into two groups: Intervention (*n* = 30) and control (*n* = 30) (Figure 1). In this way, once a lottery was drawn for the first sample to determine her group and then the next participants were assigned alternately to groups until the sample size was completed.

### Data Collection

Respondents completed two measurement instruments, including socio-demographic questionnaire (spouse and wife) and the ISA. In this study, ISA as a 30-item self-report scale was used to assess the severity of physical (ISA-P 11 items) and non-physical (ISA-NP 19 items) aggression imposed on a woman by her spouse. Responses were on a five point Likert scale from 1 to 5. A cut-off score for ISA-P scores more than 10 and ISA-NP scores more than 25 are considered to have the physical and non-physical abuse, respectively, and greater scores represent more severe abuse (25). This scale is a widely used valid and reliable self-report questionnaire (25–27). In the present study, Cronbach's alpha was 0.89, 0.80, and 0.77 for the total ISA, ISA-P and ISA-NP scores, respectively.

### Intervention

After sampling, women in the intervention group were included in the problem-solving skills training sessions to empower them in the encounter with spouse abuse. These training sessions were presented and taught by a guiding team that included a qualified psychiatric nurse (researcher) with the help and guidance of the supervisor (psychiatric nurse) and consultant (clinical psychologist). In the current study, development of the theoretical framework for the proposed intervention and training sessions was based on the review of literature and guidance of supervisor and consultant. There were 4 weekly group sessions, each session lasting about 40–50 min. The intervention group consisted of two groups with 12 women and one group with six women. The key elements and content of the problem-solving skills training sessions are shown in Table 1.

**Table 1:** Process of problem-solving skills training sessions.

**Session**	**Content**	**Duration**
First	Familiarity and communication of the members with each other and start a mutual relationship between the group leader (researcher) and the members (women) Expression of the framework and rules of participation in the group and short introduction of bipolar I disorder Expression of the role of using problem-solving skills in dealing with life problems and failures Group discussion about common problems, especially about spouse abuse Note the problems that cause spouse abuse behavior and mark one of their most important problems	40–50 min
Second	The first 10 min of the session reviewed the task given before, and group discussion about it Introduce brainstorming and ask participants to note all the solutions that they think they could have while facing their problem Group discussion about the suggested solutions for the problem	40–50 min
Third	Discuss the benefits and disadvantages of the proposed solutions by brainstorming	40–50 min
Fourth	Scoring to suggest solutions that presented by the individual based on the benefits and disadvantages and choosing the best solution Deliver and give explanations to use the problem-solving table for after the spouse's discharge	40–50 min

In the control group, the common treatment was taken for hospitalized patients and their family (abused women can talking to hospital psychologist or spouse doctor or refer to a social worker). All participants completed *pre-test* questionnaires at the beginning of the study. *Post-test* questionnaires in intervention and control groups were completed 2 months after the initial data collection. Although the training sessions were not held for the control group, after the end of the study in order to observe ethical issues, a training session (2 h) about problem-solving skills was held for this group.

### Statistical Analysis

Descriptive statistics were used to report participants' demographic and clinical characteristics. Statistical analyses were performed at a confidence level of 0.05 using IBM SPSS Statistics ver. 21.0 (IBM Co., Armonk, NY, USA). The mean and standard deviation were used to describe the quantitative variables, whereas the frequency and percentages were used to describe the qualitative variables.

The normality of the data distribution was examined using the Kolmogorov–Smirnov test. We used the non-parametric tests for the data, which were not normally distributed. Also, analysis of covariance (ANCOVA) was used to determine the effect of the intervention on abuse scores among groups.

## Results

Participant demographic characteristics and descriptive statistics are shown in Table 2. In total, 60 women of 64 participants who started the study completed the questionnaires, including 30 in the control and 30 in the intervention groups (Figure 1). Two women in the intervention group were excluded from the study because they were absent from training program more than one session (non-response rate: 3.1%). Also, two women in the control group discontinued the study because they did not complete the *post-test* assessment (non-response rate: 3.1%).

**Table 2:** Demographic and clinical data of woman experiencing bipolar spouse abuse.

	**Intervention** **(*n* = 30) M** **(SD)/*n* (%)**	**Control** **(*n* = 30) M** **(SD)/*n* (%)**	***p*-value**
Age of spouse (year)	36.4 (5.8)	33.1 (6.4)	0.04^*^
Age of women (year)	29.8 (4.2)	27.0 (4.9)	0.02^*^
Married duration (year)	5.8 (2.8)	5.0 (2.6)	0.21^*^
**Education of women**			
High school or lower	27 (90)	26 (86.7)	0.08^***^
College degree	3 (10)	7 (23.3)	
**Education of spouse**			
High school or lower	25 (83.3)	22 (73.3)	0.05^***^
College degree	5 (16.7)	8 (26.7)	
**Occupation of women**			
Employed	4 (13.3)	6 (20)	0.13^**^
Housewives	26 (86.7)	20 (66.7)	
Student	0 (0.0)	4 (13.3)	
**Occupation of spouse**			
Employed	30 (100)	30 (100)	0.34^**^
Unemployed	0 (0.0)	0 (0.0)	
Student	0 (0.0)	0 (0.0)	
**Choose a spouse**			
Voluntary	20 (66.7)	24 (80.0)	0.24^**^
Compulsory	10 (33.3)	6 (20.0)	
**Choose a wife**			
Voluntary	30 (100.0)	29 (96.7)	1.00^****^
Compulsory	0 (0.0)	1 (3.3)	
**Specific medical problem in spouse**			
Yes	11 (36.7)	9 (30.0)	0.58^**^
No	19 (63.3)	21 (70.0)	
**Type of abuse**			
Physical	10 (33.3)	11 (36.7)	1.00^**^
Non-physical	4 (13.3)	3 (10.0)	
Both	16 (53.3)	16 (53.3)	
**Spouse drug abuse duration (year)**			
Opium	5.3 ± 0.8	5.4 ± 2.0	0.90^***^
Alcohol	6.0 ± 0.0	6.7 ± 2.9	0.54^***^
Industrial drug	3.0 ± 3.8	4.0 ± 2.3	0.85^***^
Hookah and cigarettes	7.3 ± 6.2	7.3 ± 2.3	0.90^***^
Number of children	1.1 ± 9.8	1.8 ± 1.6	0.82^***^

* *Independent t-test*,

** *Chi-square test*,

*** *Mann Whitney test*,

**** *Fisher's exact test*.

Table 3 shows that a description of the women' spouse abuse scores in the intervention and control groups. In ANCOVA, the interaction between the groups and the previous values were calculated, due to the lack of interaction (such as *p* = 0.174 for age of spouse and *p* = 0.868 for age of women), the results of ANCOVA are valid, which is presented in Table 4. The results indicated that changes in abuse scores (total ISA, ISA-P, and ISA-NP) after the intervention were significantly different among the two groups (*p* < 0.0001).

**Table 3:** The description of the women' spouse abuse scores in the intervention and control groups.

**Variables**	**Group**	
	**Intervention (*n* = 30)**	**Control (*n* = 30)**
	**Means (SD)**	**Means (SD)**
**ISA-P**		
Baseline	58.1 (31.4)	52.4 (34.9)
After 2 months	42.8 (28.9)	48.2 (33.0)
**ISA-NP**		
Baseline	76.9 (10.6)	71.8 (10.0)
After 2 months	56.1 (18.1)	68.2 (13.0)
**Total ISA**		
Baseline	64.9 (21.7)	59.4 (25.0)
After 2 months	47.6 (24.1)	55.4 (24.6)

**Table 4:** Analysis of covariance of the women' spouse abuse scores in the intervention and control groups.

**Group**		**B**	**SE**	**t**	***p*-value**	**Effect size**
ISA-P	Intervention	−11.429	2.026	−5.640	0.0001	0.366
	Control					
ISA-NP	Intervention	−20.758	2.567	−8.086	0.0001	0.543
	Control					
Total ISA	Intervention	−14.557	1.967	−7.402	0.0001	0.499
	Control					

## Discussion

The aim of this study was to examine the effectiveness of psychoeducation group training based on problem-solving skills for women experiencing bipolar spouse abuse. We found that problem-solving skills training intervention can decrease the abuse (total ISA, ISA-P, and ISA-NP) experienced by a woman with a bipolar spouse. Because the hospitalized spouses in control group too received the common medical treatment for bipolar disorder, we also have a decrease in abuse scores (total ISA, ISA-P, and ISA-NP) rather than baseline time reported by women in this group. Although lower abuse scores in the intervention than control group were statistically significant.

Our results are in harmony with the findings of Maddoux et al. (23), who emphasized the use of interventions that address the problem-solving ability may be beneficial in increasing abilities of abused women to manage the daily stressors of life following abuse (23). Also, the results of studies revealed that psychoeducation intervention such as training of problem-solving skills can reduce the physical and psychological types of committed violence against pregnant women in Iran (28) and China (29). Also, as Hajian et al. (30) have reported, a high level of resilience and the use of problem-oriented coping can have a major role in reducing the likelihood of suicide attempt in Iranian females reporting spouse-related abuse (30).

Working with adolescents with bipolar mood disorder and their families (family-focused therapy, interpersonal and social rhythm therapy, cognitive-behavioral therapy, and individual or group psychoeducation) can be understood as preventive work for future relational functioning and marriage with the aim of lessening the impact of the illness (4, 31, 32).

Spouse abuse is a serious and hidden problem in societies that affects women's health and well-being. The screening and identification of women who are suffering from spouse abuse is considered to be a criterion for adequate and proper care and treatments along with a specialized referral to services. Although the studies showed that there are barriers to proper screening including lack of provider education regarding abuse, lack of time, and lack of effective interventions and patient non-disclosure (33). Among health-care professionals, nurses are in a unique position and in the first line to address this problem and, through their attention, women's health, well-being, and safety might improve (34).

### Limitations

Results of this study should be interpreted considering study limitations. In this study, we assessed the spouse abuse using the self-administered questionnaires and whether there is potential for the severity of spouse abuse to be overestimated. Thus, the use of objective measurements is recommended in future studies. Also, the individual differences of women (education, occupation, psychological and mental state, etc.) could affect the spouse abuse scores. Randomization and control group were used to minimize these differences.

### Implications for Clinical Practice

Notwithstanding these limitations, this is the first randomized controlled trial to explore the effectiveness of psychoeducation group training based on problem-solving skills on spouse abuse in Iranian women who experienced bipolar spouse abuse. Prevalence rates of spouse abuse are significantly higher in mental health disorder patients especially in Iran, but the studies in this area are limited.

Findings indicate that women with a bipolar spouse, experience a high level of physical and non-physical abuse. These result emphasized that this kind of abuse is not inevitable, and thus, must be addressed in all societies. Abuse screening for women with a mental disorder spouse needs to be incorporated as a priority in mental health services in order to reduce the morbidity and mortality issues associated with this abuse. Mental health provider especially nurses should have a holistic approach to provide care for the family of bipolar patients and develop strategies such as problem-solving skills that enable these women to contend with spouse abuse. Health professionals such as nurses that provide caring for bipolar patients and their family and have an important role in accurate identification and assessment of spouse abuse, could benefit from the results of this study.

## Conclusion

Findings of this study suggest a serious need for the accurate assessment/screening/interventions of women that live with a bipolar spouse about probable abuse and education of them by nurses, to implement appropriate and better nursing care. Providing proper interventions for the family with a mental health patient such as bipolar disorder may be forgotten by health professionals especially nurses, which suggests a need for nursing education in this area. These findings on problem-solving skills, as an essential component of cognitive-behavioral therapy that can help and enable women to manage the problematic situations of everyday life and their emotional impact from bipolar spouse abuse, and this finding can be used to implement more effective nursing education, interventions and strengthen those that are currently used.

## Data Availability Statement

The original contributions presented in the study are included in the article/supplementary material, further inquiries can be directed to the corresponding authors.

## Ethics Statement

All participants were informed about the aim and process of the study and were allowed to discontinue the study whenever they wished. Participants who were willing to participate were asked to give their informed consent. All forms and questionnaires were kept anonymous with numerical codes. This study was approved by the ethics committee of Mashhad University of Medical Sciences (Register Number: IR.MUMS.REC.1396.273) and was registered in the Iranian center of clinical trial registration with the ID number of IRCT20171223038002N1.

## Author Contributions

M-SNA: conceptualization, data curation, formal analysis, investigation, methodology, resources, software, and validation. SV: conceptualization, methodology, and supervision. SZ-A: formal analysis, resources, software, writing—original draft, writing—review, and editing. All authors contributed to the article and approved the submitted version.

## Conflict of Interest

The authors declare that the research was conducted in the absence of any commercial or financial relationships that could be construed as a potential conflict of interest.
